# Eugenol Induces Apoptosis in Tongue Squamous Carcinoma Cells by Mediating the Expression of Bcl-2 Family

**DOI:** 10.3390/life13010022

**Published:** 2022-12-21

**Authors:** Dan-Alexandru Surducan, Robert Cosmin Racea, Madalina Cabuta, Iustin Olariu, Ioana Macasoi, Laura Cristina Rusu, Sorin Dan Chiriac, Doina Chioran, Stefania Dinu, Marius Octavian Pricop

**Affiliations:** 1 Faculty of Medicine, “Victor Babes” University of Medicine and Pharmacy Timisoara, Eftimie Murgu Square No. 2, 300041 Timisoara, Romania; 2 Faculty of Dental Medicine, Victor Babeș University of Medicine and Pharmacy, 9 Revolutiei 1989 Ave., 300070 Timisoara, Romania; 3 Multidisciplinary Center for Research, Evaluation, Diagnosis and Therapies in Oral Medicine, “Victor Babes” University of Medicine and Pharmacy, Eftimie Murgu Square, No. 2, 300041 Timisoara, Romania; 4 Faculty of Pharmacy, “Victor Babes” University of Medicine and Pharmacy Timisoara, Eftimie Murgu Square No. 2, 300041 Timisoara, Romania; 5 Research Center for Pharmaco-Toxicological Evaluations, Faculty of Pharmacy, “Victor Babes” University of Medicine and Pharmacy Timisoara, Eftimie Murgu Square No. 2, 300041 Timisoara, Romania; 6 Department of Dentistry, Faculty of Dentistry, Faculty of Medicine, “Vasile Goldis” Western University of Arad, 310025 Arad, Romania; 7 Pediatric Dentistry Research Center, Faculty of Dental Medicine, Victor Babes, University of Medicine and Pharmacy Timisoara, 9 No., Revolutiei Bv., 300041 Timisoara, Romania

**Keywords:** apoptosis, eugenol, gene expression, oral cancer, RT-PCR, tongue squamous carcinoma

## Abstract

Head and neck squamous cell carcinoma is highly aggressive type of cancer for which the available treatment often causes patients severe side effects. Eugenol (Eug) is the major active constituent of clove essential oil and is known to possess antitumor properties. The present study aimed to assess the in vitro cytotoxicity of eugenol in SCC-4, tongue squamous carcinoma cells, and also in HGF, human gingival fibroblasts. Both cell lines were treated with five concentrations of Eug (0.1–1 mM) for 72 h. Cellular viability was assessed, followed by cellular morphological evaluation and by staining of the nuclei and cytoskeleton. RT-PCR was conducted in order to find the effect eugenol had on the expression on Bad, Bax, and Bcl-2 genes. Eugenol induced a dose-dependent decrease in viability in both cell lines, with the SCC-4 cells being significantly more affected. HGF cells detached from the plate at the highest concentrations used, while SCC-4 cells changed their morphology in a dose-dependent manner, with rounding, floating cells, and confluency loss being observed. Apoptotic-like signs such as chromatin and actin filaments condensation were clearly seen in SCC-4 cells, while RT-PCR revealed a significantly increased expression of pro-apoptotic genes Bax and Bad. Therefore, eugenol exerts its cytotoxic effect in tongue squamous cell carcinoma through inducing apoptosis.

## 1. Introduction

Squamous cell carcinomas (HNSCC) represent 90% of the head and neck cancers and account for 890,000 new cases each year. HNSCC is a highly aggressive type of cancer that has a 66% 5-year survival rate. The site of origin for HNSCC is the mucosal epithelium which lines the oral cavity, pharynx, larynx, and sinonasal tract, and after altering the surface epithelium, the changes progress to the subepithelial connective tissues [1,2]. Today, several risk factors are involved in the apparition of squamous cell carcinomas: chronic use of tobacco and alcohol, the latter enhancing the harmful effects of tobacco in a synergistic manner [2]. Besides chronic exposure to the aforementioned factors, several oral lesions are associated with developing HNSCC: leukoplakia, erythroplakia, and lichen planus. From these premalignant lesions, about 30% progress into cancer. While HNSCC is characterized by serious inhibition of immune competence, interestingly, the premalignant lesions show an increased level of inflammatory cytokines [3].

In order to ensure a better prognosis, it is of foremost importance to diagnose HNSCC as early as possible [4]. Presently, depending on several factors, the options for treating squamous cell carcinomas are surgery, chemotherapy, radiotherapy, and immunotherapy. Performing surgical rejection is the main treatment for malignancies in the oral cavity, but this can leave lasting effects on the speaking and swallowing capacities of the patient [5]. Radiotherapy is another treatment option, only it is associated with resistance and poor survival rates [6]. Chemotherapy with cisplatin in high doses or in combination with 5-fluorouracil has its deficiencies as well, whether we speak of developing resistance to treatment which leads to treatment failure, or of the bone marrow, gastrointestinal tract, nervous system, and urinary system toxicity [7,8]. More recent therapies are immunotherapy and targeted therapy with antibodies, but they are accompanied by immune-related adverse events [8,9]. Treatment is usually multimodal, this aspect often reduces the quality of life of the patient, and therefore, alternatives with fewer side effects are sought. Among the most intensely studied are compounds found in vegetal products, which seem to be the most promising [10].

Eugenol or 4-allyl-2-methoxyphenol is the major bioactive constituent of *Eugenia caryophyllata* (syn. *Syzygium aromaticum*) essential oil, from the *Myrtaceae* family [11]. It is an aromatic compound that belongs to the group of simple phenols, pale yellowish in color, with low solubility in water, but are well soluble in organic solvents. Eug is documented to have high antioxidant and antimicrobial activities, as well as anti-inflammatory, analgesic, and antitumor properties [12]. Some of these key effects seem to be interconnected because eugenol regulates the macrophage production of inflammatory mediators and is capable of modulating oxidative stress. Today it is known that there is a correlation between inflammation, oxidative stress, and cancer [13,14]. Currently, Eug is employed successfully in dentistry in a combination with zinc oxide (ZOE) as a capping material when the pulpotomy techniques are performed in primary teeth, precisely for its anti-inflammatory and analgesic effects. During this procedure, the affected coronal pulp tissue is removed and the amorphous chelate formed between zinc oxide and Eug is placed over the pulp tissues [15]. Eug is not only employed in the dental cabinet, but due to its antibacterial properties, it is met in oral and dental care products [12]. Moreover, it has been demonstrated that in vitro, eugenol has the ability to inhibit teeth decalcification caused by apple acidic beverages. It also may promote remineralization [16].

Eugenol is today a compound of interest, not only because of its employment in different procedures in dental medicine, but also due to its presence in several oral care products such as tooth pastes and mouthwashes. Because of its frequent usage and due to the problem oral cancer poses today, this study was conducted in order to evaluate the in vitro cytotoxicity of Eug on squamous cell tongue carcinoma. Therefore, the cytotoxicity of eugenol was assayed for its capacity to decrease cellular viability, influence cellular morphology, and to determine its impact on the structure of cell nuclei and actin filaments. In order to determine the type of cell death it induces, expressions of three pro- and anti-apoptotic genes from the Bcl-2 family were analyzed. However, because it has been stated that high doses of Eug have a cytotoxic effect on healthy human oral mucosa fibroblasts [17], our study also investigated the effect of Eug on normal human gingival fibroblasts.

## 2. Materials and Methods

### 2.1. Reagents and Culture

Eugenol, phosphate saline buffer (PBS), trypsin-EDTA solution, dimethylsulfoxide (DMSO), fetal calf serum (FCS), penicillin–streptomycin, penicillin–streptomycin–amphotericin, hydrocortisone 21-hemisuccinate sodium salt, and MTT (3-(4,5 dimethylthiazol2-yl)-2,5- diphenyltetrazolium bromide) reagent were acquired from Sigma Aldrich, Merck KgaA (Darmstadt, Germany). Fibroblast Basal Medium (ATCC^®^ PCS-201-030™), Fibroblast Growth Kit-Low Serum (ATCC^®^ PCS-201-041™), and DMEM: F-12 Medium (ATCC^®^ 30-2006™) were purchased from ATCC (American Type Culture Collection, Lomianki, Poland). Rhodamine phalloidin (00027) was acquired from Biotium (Hayward, CA, USA).

### 2.2. Cell Culture

The following cell lines were used in this study: human gingival fibroblasts—HGF (PCS-201-018™) and squamous cell carcinoma cell line—SCC-4 (CRL-1624™), both purchased from ATCC. The acquired cells came as frozen vials and were kept in liquid nitrogen. The cell lines were cultured in their specific growth medium: HGF in Fibroblast Basal Medium which was completed with the Fibroblast Growth Kit-Low Serum; 10% FCS and 1% antibiotic mixture penicillin–streptomycin–amphotericin B Solution, 0.5 mL (Final concentration penicillin: 10 Units/mL, streptomycin: 10 µg/mL, and amphotericin B: 25 ng/mL); and SCC-4 in DMEM: F12 Medium supplemented with 400 ng/mL hydrocortisone 21-hemisuccinate sodium salt, 10% FCS, and 1% penicillin (100 U/mL)–streptomycin (100 μg/mL). The cells were cultivated under standard culture conditions at 37 °C and 5% CO2 in a humidified atmosphere.

### 2.3. Cellular Viability Evaluation

Cell viability was assessed using the MTT (3-(4,5 dimethylthiazol2-yl)-2,5- diphenyltetrazolium bromide) colorimetric assay, in a similar manner as described before, according to the following protocol [18,19]. Briefly, after reaching 80% confluence, the cells were washed with PBS, detached from the culture flasks using trypsin-EDTA solution, sedimented by centrifugation and, by using trypan blue, they were counted and seeded in 96-well plates at a concentration of 1 × 10^4^/200 µL medium/well. Following adhesion, the cells were stimulated with the following concentrations of eugenol: 0.1 mM, 0.25 mM, 0.5 mM, 0.75 mM, and 1 mM. Regarding the preparation of test solutions, at first, a stock solution of eugenol solubilized in DMSO for cell culture was prepared. Then, an intermediate solution was realized using a specific growth medium. The highest concentration of DMSO used was 0.1%. After 72 h following stimulation, 10 µL/well of MTT reagent was added to each well and left for 3 h at 37 °C. For the solubilization of the MTT formazan crystals that formed, 100 μL/well of solubilization solution was added to each well. After being left 30 min at room temperature and protected from light, the absorbance was read using Cytation 5 (BioTek Instruments Inc., Winooski, VT, USA). Differences in absorbance due to the amount of formazan formed were read at 570 nm and a reference wavelength of 630 nm was used in case of background signals [19].

### 2.4. Cellular, Nuclear, and Cytoskeletal Morphology Evaluation

Microscopic examination was performed in order to visualize the impact of eugenol on cellular morphology of HGF and SCC-4 cells. The changes were observed under bright field illumination using Olympus IX73 inverted microscope (Olympus, Tokyo, Japan). The images were taken at 72 h post-stimulation and were processed using cellSens Dimensions v.17 Software (Olympus, Tokyo, Japan).

In order to provide information about the mechanisms of toxicity of eugenol on the squamous cell carcinoma cell line, the cells were subjected to DAPI and rhodamine phalloidin staining. The immunofluorescence assay was performed on the cells stimulated with 0.5 mM Eug for 72 h. Alterations in cell cytoskeletons were seen through the use of rhodamine phalloidin, while DAPI, or 4′, 6-diamidino-2-phenylindole, allowed observation of the changes in the cell nuclei [20,21]. The staining was performed in a similar manner as described before [22]. In brief, the following protocol was used: (i) the cells were seeded in 12-well plates and treated with the Eug test solutions; (ii) after 72 h, they were washed with PBS, fixed with paraformaldehyde 4%, and left 30 min at 4 °C; (iii) the paraformaldehyde was disposed of, the fixed cells were washed again with PBS and permeabilized TritonX 2% was added, and the plate was left 30 min at room temperature; (iv) TritonX 2% was disposed of and the cells were washed with TritonX 0.01% before adding blocking solution and leaving the plate for 30 min at 4 °C; (v) after the disposal of the blocking solution and the washing with TritonX 0.01%, 300 µL/well of rhodamine phalloidin were added and the plate was kept at 4 °C for 20 min; (vi) following the disposal of rhodamine phalloidin, 300 µL/well of DAPI staining was added and the plate was kept at 4 °C for 15 min; and (vii) the final step consisted of the disposal of DAPI and of washing the cells with PBS. Pictures were taken using an Olympus IX73 inverted microscope (Olympus, Tokyo, Japan) and they were analyzed with cellSens Dimensions v.17 Software (Olympus, Tokyo, Japan). The apoptotic index was calculated in order to determine the percentage of cells that present morphological changes due to apoptosis using the following formula [23]:

$$\mathrm{AI}=\frac{Number\;of\;apoptotic\;cells}{Total\;number\;of\;cells}\times 100$$

### 2.5. Gene Expression

The real-time reverse transcription–polymerase chain reaction (RT-PCR) method was used to obtain a more detailed understanding of the possible mechanisms involved in eugenol-induced cell death. In this study, the following genes were analyzed: Bax, Bcl-2 (Thermo Fisher Scientific, Inc., Waltham, MA, USA), and Bad (Eurogentec, Seraing, Belgium). The cells were prepared by culturing them in 6-well plates at a density of 1 × 10^6^ cells per well. As soon as the cells reached a state of optimal confluence, they were tested with a concentration of 0.5 mM eugenol. Total RNA was isolated using Trizol reagent and Quick-RNA^TM^ purification kit. The RNA concentration was measured using DS-11 spectrophotometer (DeNovix, Wilmington, DE, USA). After reverse transcription of RNA using the Maxima^®^ First Strand cDNA Synthesis Kit, RT-PCR quantification was conducted using the Quant Studio 5 real-time PCR system (Thermo Fisher Scientific, Inc., Waltham, MA, USA) in the presence of the Power SYBR-Green PCR Master Mix (Thermo Fisher Scientific, Inc., Waltham, MA, USA).

### 2.6. Statistical Analysis

Data are presented as means ± standard deviation (SD), with the tests performed being one-way ANOVA test followed by Dunnett’s multiple comparisons post hoc test. GraphPad Prism version 9.4.0 for Windows (GraphPad Soft-ware, San Diego, CA, USA, www.graphpad.com) was used for analyzing data. The statistically significant differences are labeled with * (* *p* < 0.1; ** *p* < 0.01; *** *p* < 0.001; **** *p* < 0.0001).

## 3. Results

### 3.1. Cell Viability Evaluation

In order to assess the viability of human gingival fibroblasts (HGF) and of squamous cell carcinoma cells (SCC-4) after 72 h of stimulation with 5 different concentrations of eugenol, 0.1 mM, 0.25 mM, 0.5 mM, 0.75 mM, and 1 mM, the MTT assay was performed.

**Figure 1:**
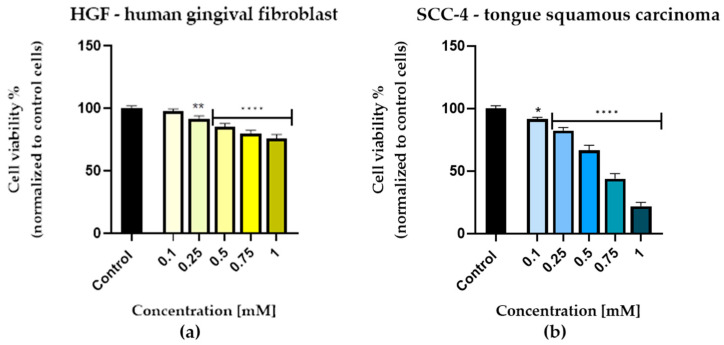
In vitro cell viability evaluation of eugenol (0.1 mM, 0.25 mM, 0.5 mM, 0.75 mM, and 1 mM) in HGF (human gingival fibrolasts) (**a**) and in SCC-4 (tongue squamous cell carcinoma) (**b**). MTT colorimetric assay was performed after 72 h treatment. Results are presented as viability percentages (%) normalized to control (DMSO-treated cells). Presented data are expressed as mean values ± SD of three independent experiments performed in triplicate. One-way ANOVA test was applied in order to see the statistical differences between the control and the treated group followed by Dunnett’s multiple comparisons post hoc test. “*” marks the statistical differences between data (* *p* < 0.05; ** *p* < 0.01; and **** *p* < 0.0001).

Figure 1 shows a concentration-dependent decrease in viability in both cell lines. The healthy human gingival fibroblasts were not significantly affected by the 0.1 mM concentration, but a discrete dose-dependent loss of viability was seen at higher concentrations (0.5 mM, 0.75 mM, and 1 mM). Nevertheless, even at a concentration of 1 mM, cell viability did not drop below 50%, with an approximate value of 76% (Figure 1a). In the SCC-4 cell line, the 72 h treatment produced a concentration-dependent decrease in viability compared with the control cells. Consequently, the viability of cells did not decrease significantly at the lowest concentration tested (0.1 mM), with the viability being approximately 91% in this case. In contrast, the higher concentrations resulted in a marked reduction in cell viability, with the most significant effect observed at 1 mM, where the cell viability reached approximately 19% (Figure 1b).

### 3.2. Cellular, Nuclear, and Cytoskeletal Morphology Evaluation

To see how the test solutions impact HGF and SCC-4 cell lines, morphology analysis of the cells was performed. Human gingival fibroblasts are adherent, spindle-shaped cells. At low concentrations of Eug (0.1 mM and 0.25 mM), no significant changes were seen in their morphology, with the tested compound having no negative impact at these concentrations on the confluency of the cells or on their capacity to adhere to the plate. On the other hand, at a concentration of 1 mM, detachment from the plate and rounded cells were noticed. Bright field illumination images of the morphology of HGF cells are presented in the Supplementary Materials, Figure S1.

Under normal culture conditions, human squamous carcinoma cells are adherent, epithelial-like cells, but after 72 h of treatment with Eug, dose-dependent morphological changes were seen, such as rounding and floating cells. The higher the concentration used, the more prominent the morphology changes were, with 0.75 mM and 1 mM concentrations affecting cells the most. Rounded and shrunken cells which lost contact with the neighboring cells were noticed. Moreover, floating cells were seen as a sign of plate detachment and confluency loss. Representative images are presented in Supplementary Materials, Figure S2.

**Figure 2:**
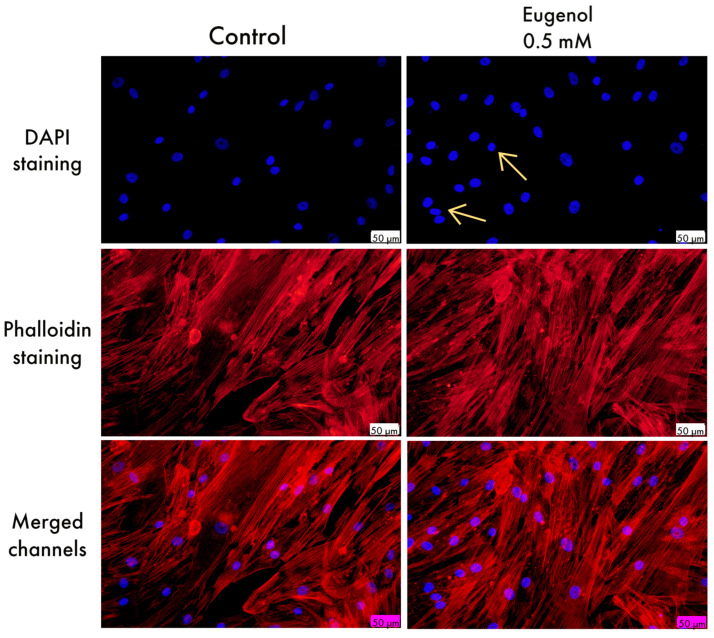
HGF cells visualized by fluorescence microscopy after treatment with Eug 0.5 mM. The impact of Eug at the level of: nuclei—DAPI staining (blue) and F-actin fibers—rhodamine phalloidin (red). Discrete nuclear condensation can be noticed in case of the treated cells, marked with arrows. The scale bar indicates 50 μm.

Using fluorescence microscopy, nuclear and cytoskeletal changes in human gingival fibroblasts and squamous carcinoma cells were analyzed after treatment with 0.5 mM Eug. The untreated HGF cells showed no nuclear or cytoskeletal changes under fluorescence microscopy, while in the Eug-treated cells, a slight nuclear condensation was noticed (Figure 2).

**Figure 3:**
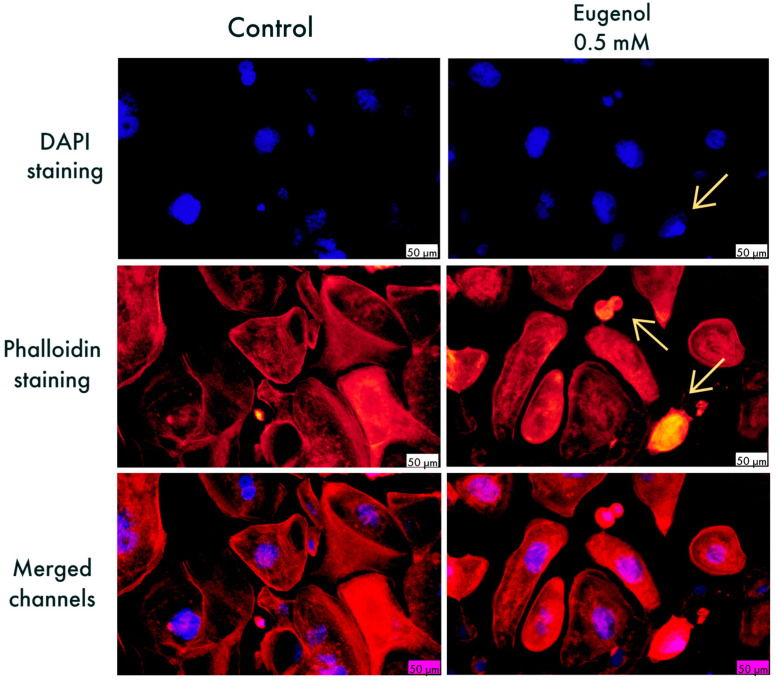
SCC4 cells visualized by fluorescence microscopy, after treatment with Eug 0.5 mM. The impact of Eug at the level of: nuclei—DAPI staining (blue) and F-actin fibers—rhodamine phalloidin (red). Nuclear fragmentation, chromatin, and peripheral actin fiber condensation can be seen in the treated cells, marked with arrows. The scale bar indicates 50 μm.

**Figure 4:**
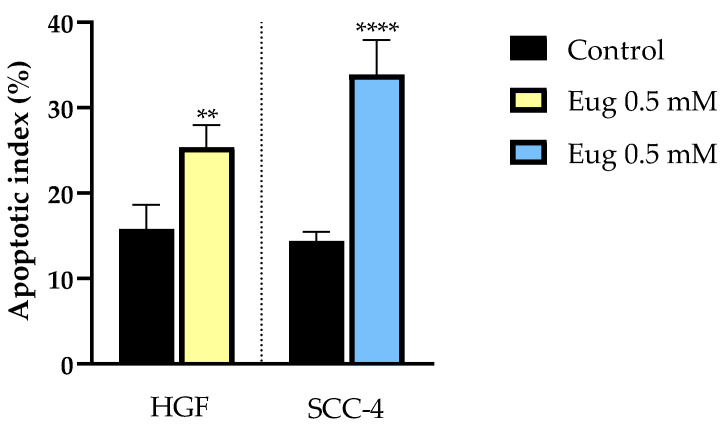
Apoptotic index (AI) determination in DAPI-stained HGF and SCC-4 cells following 72 h treatment with 0.5 mM Eug. Results are presented as apoptotic index (%) normalized to control. Data are expressed as mean values ± SD of three independent experiments performed in triplicate. One-way ANOVA test was applied in order to see the statistical differences between the control and the treated group followed by Dunnett’s multiple comparisons post hoc test. “*” marks the statistical differences between data (** *p* < 0.01 and **** *p* < 0.0001).

Eug-treated SCC-4 cells stained with DAPI showed signs of chromatin condensation and fragmentation inside the nuclei. The organization of actin fibers suffered significant changes after treatment with the 0.5 mM concentration of Eug. In the cells stained with rhodamine phalloidin, condensation of actin fibers, especially at the periphery and cell rounding, was noticed. Morphological evaluation was conducted in comparison with control cells where no significant changes were seen (Figure 3). According to the apoptotic index calculation, Eug exerted a pro-apoptotic effect in both cells, HGF and SCC-4, with a higher value of AI in SCC-4 (Figure 4).

### 3.3. Gene Expression

In previous evaluations it was observed that eugenol causes changes characteristic of apoptosis at the level of cell morphology and nucleus structures as well as cytoskeleton organization. Therefore, the next objective of the study was to examine the main genes involved in apoptosis (Bax and Bad—pro-apoptotic genes and Bcl-2—anti-apoptotic gene).

**Figure 5:**
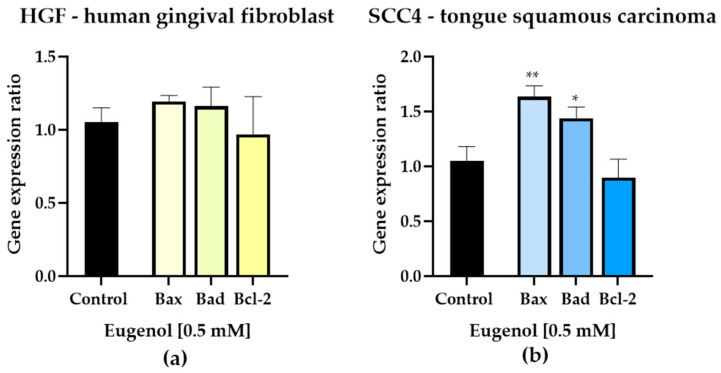
Relative fold change expression of mRNA of proapoptotic (Bax and Bad) and anti-apoptotic (Bcl-2) markers in human gingival fibroblast (HGF) (**a**) and in tongue squamous carcinoma cells (SCC-4) (**b**) 72 h after exposure to Eug 0.5 mM. mRNA expression levels normalized to 18 S expression. Mean values ± SD of three independent experiments are presented. One-way ANOVA with Dunnett’s multiple comparisons post hoc test was used to identify the statistical differences (* *p* < 0.05 and ** *p* < 0.01).

After testing an intermediate concentration of Eug (0.5 mM) in healthy gingival fibroblast cells, the results indicated that eugenol induces a slight increase in pro-apoptotic genes, especially the Bax gene. Bcl-2 gene expression, however, was not significantly altered compared with the control cells (Figure 5a).

In tongue squamous carcinoma cells, the sub-toxic dose of Eug, 0.5 mM, significantly increased the expression of mRNAs for the pro-apoptotic markers—Bax and Bad, the most significant increase was recorded for the expression of Bax. Furthermore, Eug had no significant effect on the expression of Bcl-2, an anti-apoptotic gene (Figure 5b).

## 4. Discussion

In the past years, many advances were made in the field of cancer treatment; however, cancer is a great burden worldwide due to its high incidence and mortality [24]. Head and neck squamous cell carcinoma is a highly aggressive type of cancer, which represents 90% of the head and neck cancers and accounts for 890,000 new cases each year [1,2]. Treatment is usually multimodal, and the often severe side effects are reducing considerably the patient’s life quality [8]. Therefore, better alternatives are sought for. Answers have been found in the kingdom Plantae, as natural compounds have been shown to inhibit cancer development. The ways through which phytochemicals produce the anticancer effect are numerous, with inducing cell apoptosis being just one of them [10]. One such compound that has triggered the interest of researchers is eugenol, a naturally occurring compound found especially in clove essential oil, is known today for its various properties: antioxidant, anti-inflammatory, analgesic, and antimicrobial, for its employment in dental medicine [11,13]. Until now, Eug has been shown to induce apoptosis and to inhibit metastasis in various cancer cell lines and is considered a potent natural candidate in the treatment of cancer [12].

In light of these premises, the purpose of the present study was to evaluate Eug’s antitumor potential at the level of tongue squamous carcinoma cells. For a more thorough evaluation of the cytotoxic potential of Eug, a healthy cell line (gingival fibroblasts) was also selected. To determine the cytotoxic potential, MTT assay was used as a colorimetric method for determining cellular viability. According to the results, cell viability decreased slightly in the case of HGF cells, with the highest concentration causing a decrease in cell viability up to a value of approximately 76%. According to ISO Standard 10993-5:2009, a compound is cytotoxic if it affects the cells in a proportion of more than 30% [25]. Thus, Eug does not appear to predict a cytotoxic effect on gingival fibroblasts. The tongue cancer cell line, SCC-4, was significantly affected by the 72 h treatment with 0.1–1 mM eugenol. A concentration-dependent loss of viability was seen in comparison with the control. The MTT assay is a cell viability colorimetric assay, commonly met in research, based on the principle that the metabolic active cells, through dehydrogenase enzyme, reduce the tetrazolium salt to formazan, an insoluble molecule, which is further solubilized by the addition of a solubilizing agent [26]. Based on the intensity of the colored formazan product, which has an absorbance near 570 nm, the assay enabled us to assess the viability of cells treated with various concentrations of Eug. The concentrations used in this study (0.1 mM, 0.25 mM, 0.5 mM, 0.75 mM, and 1 mM) were chosen based on a comprehensive review of the currently available literature [27,28,29,30]. Several in vitro studies have evaluated eugenol’s cytotoxicity in blood, breast, skin, cervical, and colon cell lines via various mechanisms of action [31]. One suggestive example is the study conducted by Jaganathan S. K. and collaborators, who tested Eug on colon carcinoma cell lines HCT-15 and HT-29, revealing through the MTT assay an inhibitory effect in both cancer cell lines at an IC50 of 300 μM and 500 μM, with Eug also producing DNA fragmentation [29]. These results show that our cancer cell toxicity findings are in accordance with the ones reported in the literature.

Moreover, due to its usage in dentistry and because of the aggressiveness and invasiveness of oral squamous cell carcinomas, it was also tested for its cytotoxic effect on oral cancer cells. While the present study chose SCC-4 tongue squamous cancer cell line, the study conducted by Kim Y. and Park B, tested eugenol at concentrations of 0.5 mM, 1 mM, and 2 mM in HSC-2, an oral cavity squamous cell carcinoma cell line, showing Eug’s cytotoxicity towards it through inducing apoptosis [27]. On the other hand, one of the earliest mentions of the cytotoxicity eugenol has on human oral mucosal fibroblast dates from 1994, when Jeng J.H. and his collaborators showed that Eug concentrations higher than 3 mmol/L elicited a concentration- and time-dependent cytotoxic effect [17]. The concentrations of Eug used for the current study impacted the human fibroblasts in a concentration-dependent manner, but the viability did not decrease below 76%.

The morphology of the cells after treatment with Eug was assessed in order to determine the extent of its cytotoxicity. In the case of HGF cells, Eug determined the appearance of morphological changes in a dose-dependent manner. Therefore, at the lowest tested concentrations (0.1 mM and 0.25 mM), no significant morphological changes were observed. In contrast, higher concentrations, and specifically the concentration of 1 mM, led to morphological changes such as rounding of the cells and a decrease in their confluency. Aligning with the cellular viability results, the visualization of Eug-treated SCC-4 cells under bright-field illumination microscopy showed rounded and floating cells, loss of contact with the neighboring cells, and also confluence reduction in a dose-dependent manner. It is known today that extrusion from the cell monolayer is a sign of apoptosis [32]. Similar results were obtained by Júnior P. and colleagues who have stimulated two breast cancer cell lines (MCF-7, MDA-MB-231), a cervix cancer cell line (SIHA), and two melanoma cell lines (SK-Mel-28 and A2058) with various concentrations of Eug. Their results showed a concentration-dependent loss of viability, accompanied by morphological changes and plate detachment—all seen through light and electron microscopy analysis [33].

Staining studies were conducted for the purpose of gaining a better understanding of the potential mechanism of action linked to eugenol. They were conducted at a subtoxic concentration (0.5 mM) at which a decrease in cell viability was observed, but not to a great extent. For the present study, DAPI was used for following the alterations in the nuclei of treated and untreated cells and rhodamine phalloidin for staining actin filaments. Slight condensation of some cell nuclei were seen in HGF cells (Figure 2), in comparison with SCC-4, where visible apoptotic bodies and condensation of chromatin were seen (Figure 3). The visualization of the nuclei showed more intense fluorescence in the condensed nuclei, besides rounding and shrinkage in their size. Moreover, following stimulation with Eug, the immunofluorescence assay revealed reorganization of actin filaments in SCC-4 cells under the shape of peripheral condensation ring.

The decrease in cellular viability can be explained by various forms of cell death, among which apoptosis has been the most intensely studied [32]. According to the available literature, apoptosis can be recognized by specific morphological changes which can be seen under brightfield illumination and fluorescence microscopy altogether. Firstly, the apoptotic cell starts rounding up and partially detaches from the extracellular matrix [34]. The cell becomes smaller in size, due to the dense package of the cell contents. At a nuclear level, pyknosis appears, which means the chromatin is irreversibly condensed, followed by karyorrhexix or nuclear fragmentation [35]. At a cytoskeletal level, a peripheral ring is formed by the actin which suffers reorganization. After phosphorylation of myosin II, the contraction of the cortical ring takes place and due to the difference in the pressure gradient between the interior of the cell and exterior, membrane blebbing appears [34]. Another sign of apoptosis is the formation of apoptotic bodies, made of cytoplasm, organelles, and nuclear content [36]. Chromatin and nuclear matrix can easily be seen by fluorescence microscopy through staining of cells with a DNA-binding fluorochrome, one such fluorescent dye being DAPI, or 4′, 6-diamidino-2-phenylindole, which binds to the AT-rich regions of DNA. The blue fluorescence of DAPI permits the visualization of nuclear fragments and condensed nuclei, with the advantages of using it being DNA selectivity and enhanced cell permeability [20]. Moreover, the cell’s cytoskeleton is formed of actin filaments, microtubules, and intermediate filaments, constituents which are important to ensure cell motility, plasma integrity, characteristic cell morphology, and for allowing cellular transport [37]. However, one of the hallmarks of cancer is exactly the increased motility of cells, which allows the invasion of adjacent healthy tissues and metastasis [38]. Actin, a protein abundant in the cytoskeleton, is vital for cell motility and hence is a molecule that has the ability to produce changes in actin’s structure and can be a potential tool in fighting cell cancer progression. As specified above, in order for a cell to undergo apoptosis, remodeling of the cell is necessary, with polymerized actin playing a major role in the process [34]. Through the staining of cytoskeletal elements, fluorescence microscopy allows the observance of architectural structure and changes, with rhodamine phalloidin being one example that allows the visualization of F-actin and the changes that may occur [21]. It is known that cancerous cells have the ability to escape apoptosis in order for the tumor to progress [39]. Studies have shown that targeting these apoptotic pathways can be a key tool in cancer therapy [40]. Our study revealed that eugenol induces the above-mentioned morphological changes in oral squamous carcinoma cells: rounding and shrinkage of cells, chromatin condensation, formation of apoptotic bodies, and peripheral actin condensation; therefore, causing apoptotic-like effects.

In order to further analyze the mechanism that lays behind the cell death, a study of eugenol’s ability to produce changes in pro- and anti-apoptotic gene expression was conducted. The process of apoptosis can be initiated through two pathways: an intrinsic one, known as the mitochondrial pathway, or an extrinsic one [41]. Mitochondria release pro-apoptotic proteins which are responsible for DNA fragmentation and chromatin condensation into the cytoplasm. The permeabilization of their membranes is controlled by the BCL-2 family of proteins [36]. The aforementioned family of proteins contain anti-apoptotic proteins (such as BCL-2), pro-apoptotic proteins (such as BAX), and (BH-3)-only proteins which regulate the first two subfamilies (such as BAD) [41,42]. Once the pro-apoptotic proteins are activated, or anti-apoptotic ones are suppressed, apoptosis is initiated [35]. Through real-time reverse transcription–polymerase chain reaction, the expressions of Bax and Bad, two pro-apoptotic genes, and of Bcl-2, an anti-apoptotic gene were analyzed. A slight increase in the expression of Bax and Bad genes was obtained in the case of HGF cells; whereas, the expression of the anti-apoptotic gene Bcl-2 was not significantly modified. In the case of SCC-4 cells, treatment with a sub-toxic dose of Eug significantly increased the expression of pro-apoptotic markers Bax and Bad. These findings are aligning with the available literature on this topic. Ghodousi-Dehnavi E. et al. showed that Eug has the ability to increase the expression of the p53 tumor suppressor gene and decrease the expression of the KRAS oncogene gene in colon adenocarcinoma cells [30]. Moreover, Fathy M. and colleagues showed that Eug produces a decrease in the expression of the Bcl-2 gene, while increasing the expression of the Bax gene in HeLa cells [43]. These findings showed that eugenol is capable of inducing apoptosis through the intrinsic pathway. These studies are in correlation and support the results presented in the current study.

## 5. Conclusions

The present study focused on evaluating the in vitro cytotoxic effect of eugenol, the most abundant constituent of *Eugenia caryophyllata* volatile oil, on oral squamous carcinoma cells, as well as at the level of human gingival fibroblasts. According to the results, eugenol inhibits tongue squamous carcinoma cells in a dose-dependent manner. Additionally, the cytotoxic effect was associated with the appearance of apoptotic-like changes in cell morphology, as well as in nuclei and cytoskeleton structures. Furthermore, Eug treatment of SCC-4 cells resulted in an increase in the expression of pro-apoptotic markers—Bax and Bad. These findings suggest that eugenol may have potential as an antitumor agent, but further investigations are required to elucidate the biological mechanisms underlying its antitumor properties.

## Data Availability

Not applicable.

## Supplementary Materials

The following supporting information can be downloaded at: https://www.mdpi.com/article/10.3390/life13010022/s1, Figure S1: Bright field illumination images of the morphology of HGF cells after 72 h treatment with eugenol 0.1–1 mM. Detachment from the plate and rounded cells can be noticed at 1 mM, marked with arrow. The scale bar indicates 50 μm; Figure S2: Bright field illumination images of the morphology of HGF cells after 72 h treatment with eugenol 0.1–1 mM. Rounded and shrinked cells which have lost contact with the neighbouring cells can be seen, marked with arrows. The scale bar indicates 50 μm.
