# Supplementary material for: Eugenol Induces Apoptosis in Tongue Squamous Carcinoma Cells by Mediating the Expression of Bcl-2 Family
Source: Life (Basel). 2022 Dec 21;13(1):22. doi: 10.3390/life13010022 (PMC9861585; doi:10.3390/life13010022)
Supplement: Supplementary file 1 [file life-13-00022-s001.zip › life-2089387-supplementary.pdf]

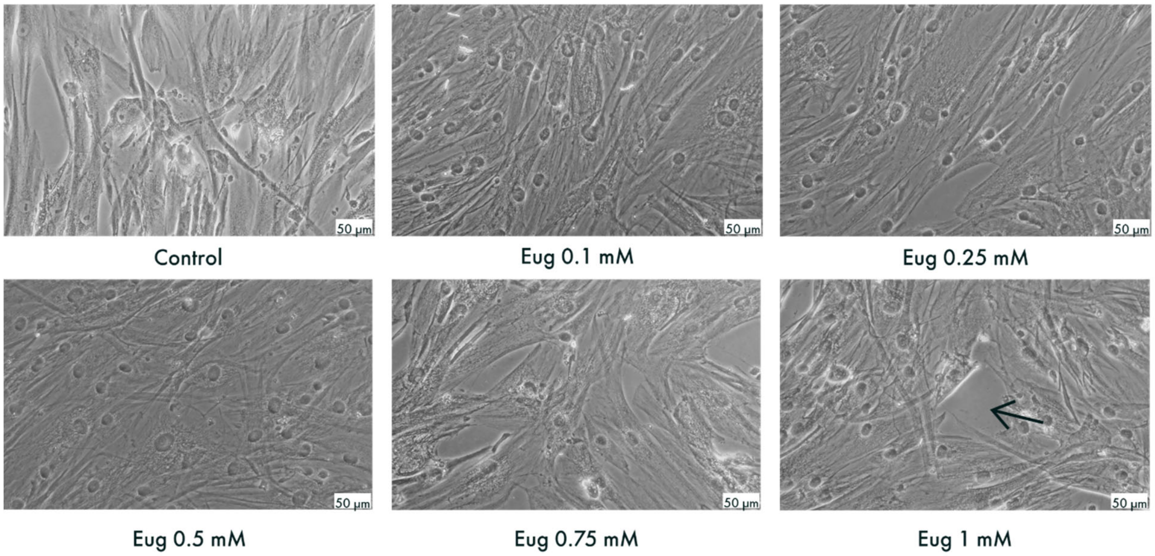

Figure S1. Bright field illumination images of the morphology of HGF cells after 72h treatment with eugenol 0.1-1 mM. Detachment from the plate and rounded cells can be noticed at 1 mM, marked with arrow. The scale bar indicates 50 μm.

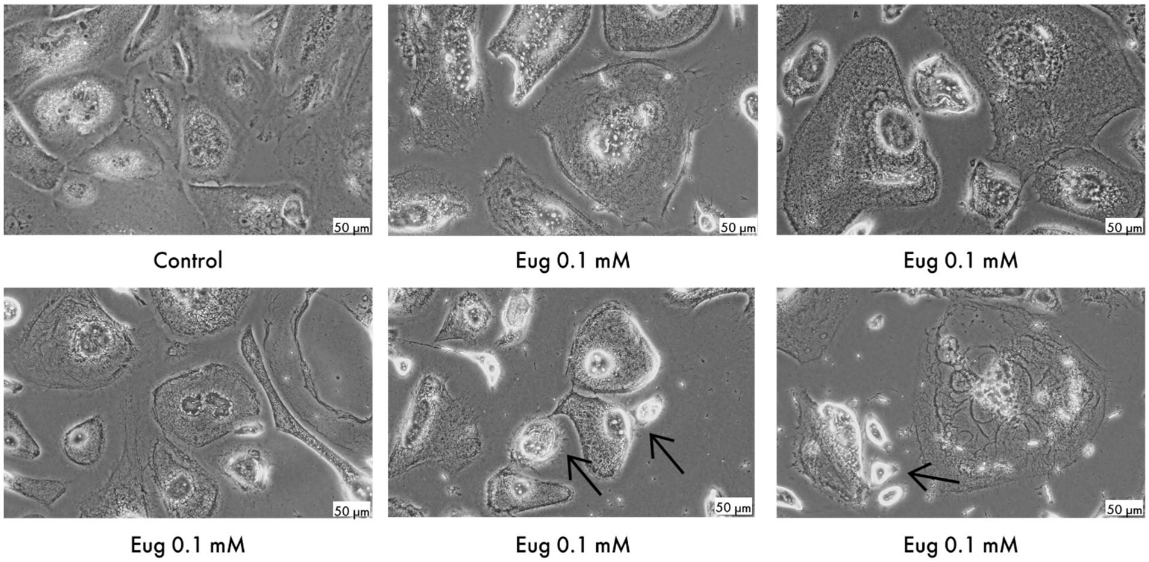

Figure S2. Bright field illumination images of the morphology of SCC-4 cells after 72h treatment with eugenol 0.1-1 mM. Rounded and shrinked cells which have lost contact with the neighbouring cells can be seen, marked with arrows. The scale bar indicates 50 μm.
